# Supplementary material for: Overexpressing GH3.1 and GH3.1L reduces susceptibility to Xanthomonas citri subsp. citri by repressing auxin signaling in citrus (Citrus sinensis Osbeck)
Source: PLoS One. 2019 Dec 12;14(12):e0220017. doi: 10.1371/journal.pone.0220017 (PMC6907806; doi:10.1371/journal.pone.0220017)
Supplement: S1 Fig — (DOCX) [file pone.0220017.s001.docx]

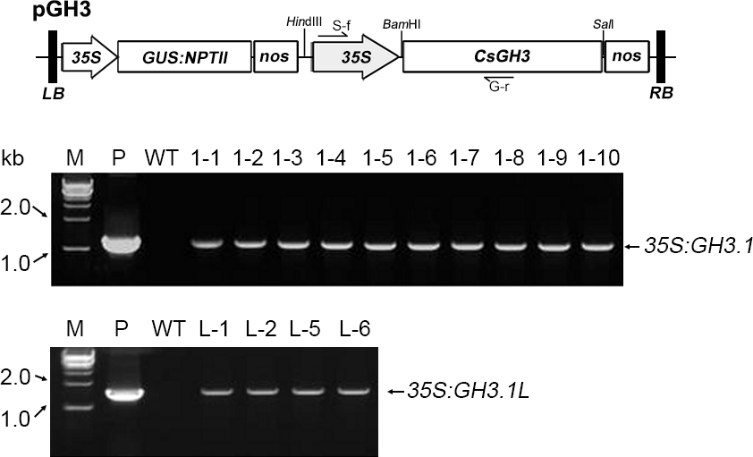


**S1 Fig.** Production of transgenic citrus plants. (**a**) Plant expression vectors used for citrus transformation. *35S*, Cauliflower mosaic virus *35S* promoter from tobacco; *gus::npt-II*, fusion of β-glucuronidase and neomycin phosphotransferase genes (for screening of transgenic plants); *nos*, nos terminator; LB, left border; RB, right border. (**b**) PCR confirmation of transgenic citrus plants. Primers S-f/G-r (35S-f/GH3.1-r and /GH3.1L-r) were used to detect 35S:GH3.1and 35S:GH3.1L expression cassettes in GUS-positive transgenic shoots, respectively. Lane M, DNA molecular size marker; lane P, plasmid template; lane WT, template from wildtype.
