# Supplementary material for: Overexpressing GH3.1 and GH3.1L reduces susceptibility to Xanthomonas citri subsp. citri by repressing auxin signaling in citrus (Citrus sinensis Osbeck)
Source: PLoS One. 2019 Dec 12;14(12):e0220017. doi: 10.1371/journal.pone.0220017 (PMC6907806; doi:10.1371/journal.pone.0220017)
Supplement: S2 Fig — (DOCX) [file pone.0220017.s002.docx]

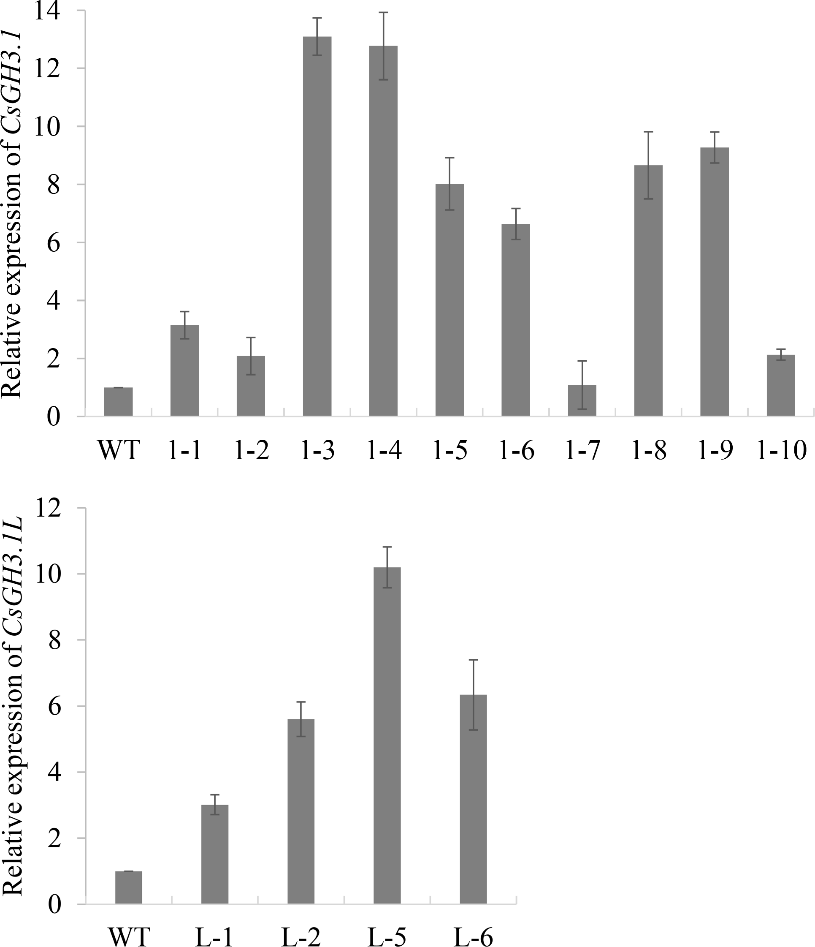


**S2 Fig.** Quantitative PCR analysis of the expression of *GH3.1* and *GH3.1L* in transgenic plants. Total RNAs was isolated from leaf tissues. Relative expressions of transgenes in transgenic plants were calculated compared with wild type plants. WT, wildtype. Error bars indicate standard deviation values of three independent tests.
