# Supplementary material for: Overexpressing GH3.1 and GH3.1L reduces susceptibility to Xanthomonas citri subsp. citri by repressing auxin signaling in citrus (Citrus sinensis Osbeck)
Source: PLoS One. 2019 Dec 12;14(12):e0220017. doi: 10.1371/journal.pone.0220017 (PMC6907806; doi:10.1371/journal.pone.0220017)
Supplement: S3 Fig — (DOCX) [file pone.0220017.s003.docx]

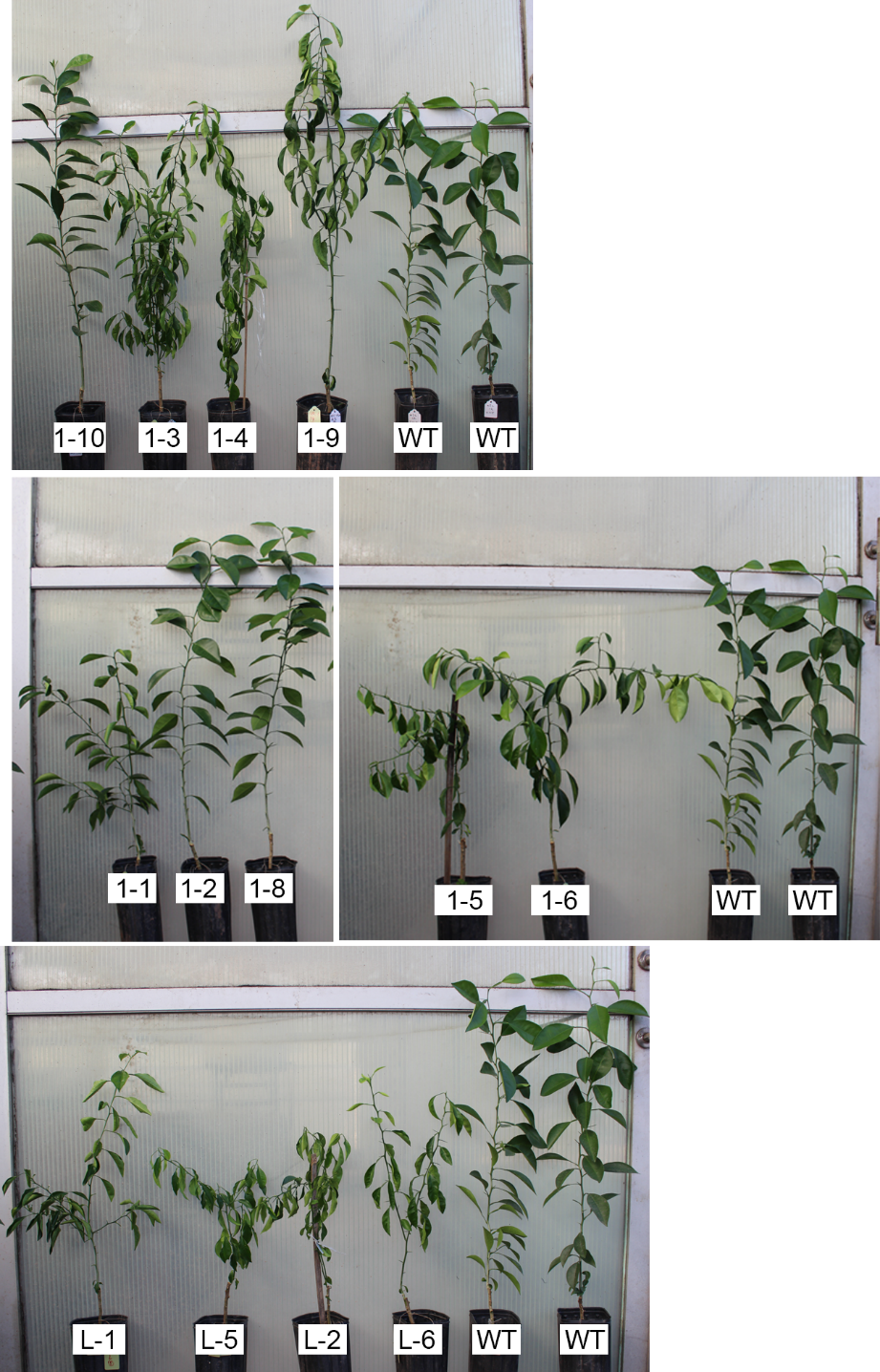


**S3 Fig.** Phenotype analysis of transgenic citrus plants overexpressing *CsGH3.1* or *CsGH3.1L* growing for one year in the greenhouse.
