# Supplementary figures and images for: Overexpressing GH3.1 and GH3.1L reduces susceptibility to Xanthomonas citri subsp. citri by repressing auxin signaling in citrus (Citrus sinensis Osbeck)
Source: PLoS One. 2019 Dec 12;14(12):e0220017. doi: 10.1371/journal.pone.0220017 (PMC6907806; doi:10.1371/journal.pone.0220017)

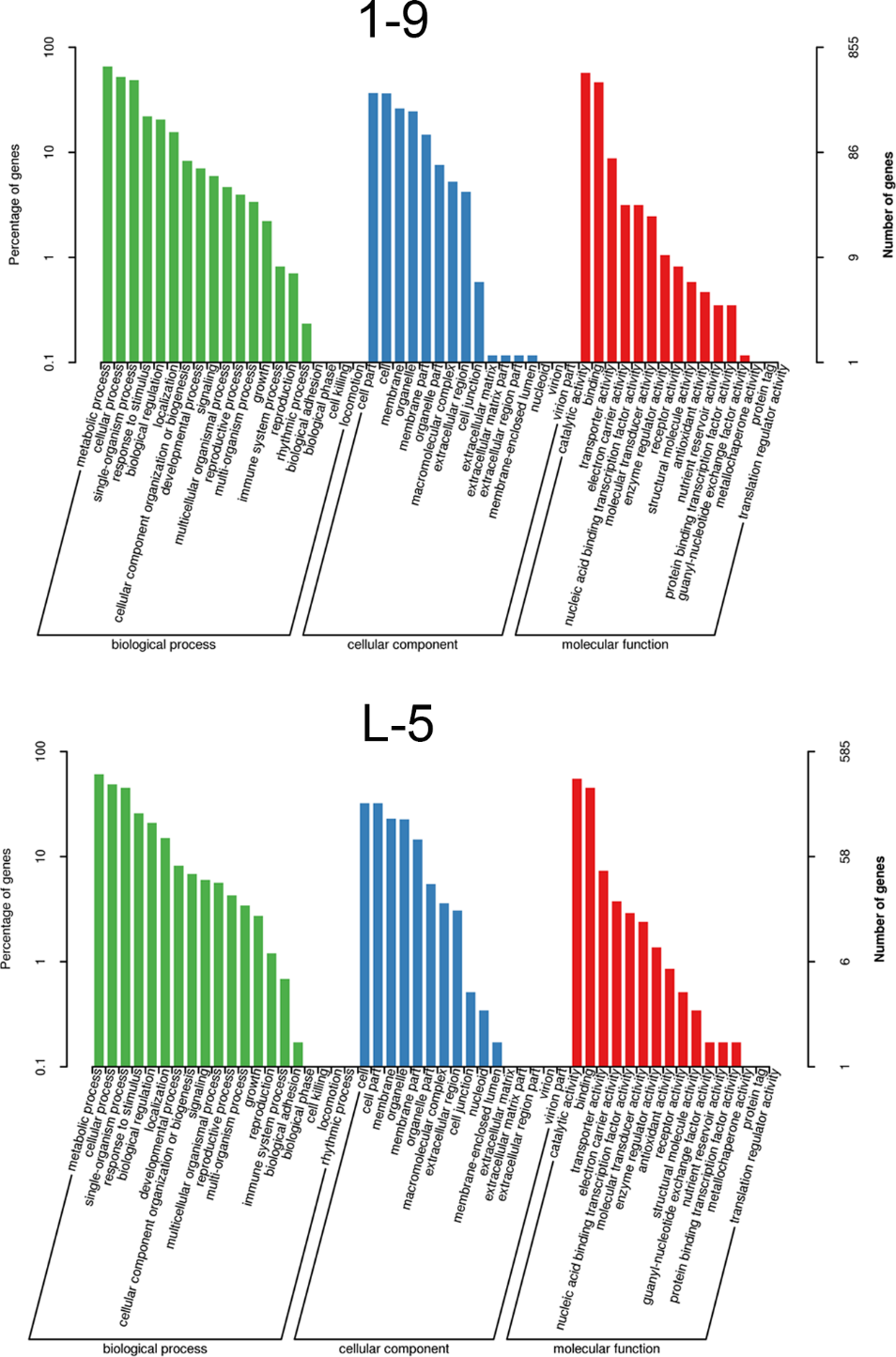


**S5 Fig.** GO classification map of the differentially expressed genes in transgenic plants.

Supplement: S5 Fig — (DOCX) [file pone.0220017.s005.docx]
