# Supplementary material for: Overexpressing GH3.1 and GH3.1L reduces susceptibility to Xanthomonas citri subsp. citri by repressing auxin signaling in citrus (Citrus sinensis Osbeck)
Source: PLoS One. 2019 Dec 12;14(12):e0220017. doi: 10.1371/journal.pone.0220017 (PMC6907806; doi:10.1371/journal.pone.0220017)
Supplement: S1 Table — (DOCX) [file pone.0220017.s006.docx]

**S1 Table.** The primers used in the study

| Name | Sequence (5ˊ-3ˊ) | Purpose |
| --- | --- | --- |
| 35S-f | GGAGTCAAAGATTCAAATAGAGGACCTAAC | PCR confirmation for the integration of transgenes in transgenic plants |
| GH3.1-r | TAGTAGCTTGTGAGCACCGGACGT |  |
| GH3.1L-r | GCCATTGGTAATTTGCCTCCGCTGTA |  |
| qGH3.1-f | TGAGTTCTTGCCTCACGACC | Quantitative RT-PCR analysis for expression of target genes |
| qGH3.1-r | TGGGGAGCCGAGTTGTAGTA |  |
| qGH3.1-r | TTGATCTCAAGCGGCCCAAT |  |
| qGH3.1L-f | TGAGTTTCTTCCCCACGAGC |  |
| qGH3.1L-r | TACAAGCCGGCGTAAGTTGT |  |
| qActin-f | CATCCCTCAGCACCTTCC | Reference gene for gene expression analysis |
| qActin-r | CCAACCTTAGCACTTCTCC |  |
