# Supplementary material for: Overexpressing GH3.1 and GH3.1L reduces susceptibility to Xanthomonas citri subsp. citri by repressing auxin signaling in citrus (Citrus sinensis Osbeck)
Source: PLoS One. 2019 Dec 12;14(12):e0220017. doi: 10.1371/journal.pone.0220017 (PMC6907806; doi:10.1371/journal.pone.0220017)
Supplement: S3 Table — (DOCX) [file pone.0220017.s008.docx]

**S3 Table.** Statistic analysis of different expression genes (DEG) in transgenic plants

| Line | No. of DEG | No. of up-regulated gene | No. of down-regulated gene |
| --- | --- | --- | --- |
| 1-9 | 1560 | 834 | 726 |
| L-5 | 1037 | 619 | 418 |
